# Effect of intrathecal lipophilic opioids on the incidence of shivering in women undergoing cesarean delivery after spinal anesthesia: a systematic review and bayesian network meta- analysis of randomized controlled trials

**DOI:** 10.1186/s12871-020-01116-5

**Published:** 2020-08-26

**Authors:** Yamini Subramani, Mahesh Nagappa, Kamal Kumar, Lee-Anne Fochesato, Moaz Bin Yunus Chohan, Yun Fei Zhu, Kevin Armstrong, Sudha (Indu) Singh

**Affiliations:** 1 Department of Anesthesia and Perioperative Medicine, Schulich School of Medicine, & Dentistry, Western University, London Health Sciences Centre- University Hospital, (LHSC-UH) , London, Ontario, Canada; 2 Department of Anesthesia and Perioperative Medicine, London Health Sciences Centre and St. Joseph’s Health Care, Western University, London, Ontario, Canada; 3 Department of Anesthesia and Perioperative Medicine, Schulich School of Medicine, & Dentistry, Western University, London Health Sciences Centre- Victoria Hospital, (LHSC-VH) , London, Ontario, Canada

## Abstract

**Background:**

Shivering is a common side effect in women having cesarean delivery (CD) under spinal anesthesia, which can be bothersome to the patient, and it can also interfere with perioperative monitoring. In several studies, the intrathecal (IT) addition of a lipophilic opioid to local anesthetics has been shown to decrease the incidence of shivering.

**Objective:**

We performed this network meta-analysis to evaluate the effects of intrathecal lipophilic opioids in preventing the incidence of shivering in patients undergoing CD.

**Methods:**

This review was planned according to the PRISMA for Network Meta-Analysis (PRISMA-NMA) guidelines. An English literature search of multiple electronic databases was conducted. We included randomized controlled trials (RCTs) that reported on the incidence of shivering, with study groups receiving either IT fentanyl, sufentanil, or meperidine in women undergoing CD under spinal anesthesia. Quality of the studies was assessed using the modified Oxford scoring system. Using random-effects modeling, dichotomous data were extracted and summarized using odds ratio (OR) with a 95% credible interval (CrI). Statistical analysis was conducted using R studio version 1.0.153 - Inc.

**Results:**

Twenty-one studies consisting of 1433 patients (Control group: 590 patients in twenty-one studies; Fentanyl group:199 patients in seven studies; Sufentanil group: 156 patients in five studies; Meperidine group: 488 patients in ten studies) met the inclusion criteria for this systematic review investigating the effect of intrathecal lipophilic opioids in preventing the incidence of shivering in women undergoing cesarean delivery under spinal anesthesia. Methodological validity scores ranged from 3 to 7. The Bayesian mixed network estimate showed the incidence of shivering was significantly lower with IT fentanyl (pooled odds ratio (OR): 0.13; 95% credible interval (CrI): 0.04 to 0.35; *P* = 0.0004) and IT meperidine (OR: 0.12; 95% CrI: 0.05 to 0.29; *P* < 0.00001), but not with IT sufentanil (OR: 0.37; 95% CrI: 0.11 to 1.22; *P* = 0.23). The IT fentanyl group had a significantly lower incidence of intraoperative discomfort [Risk Ratio (RR): 0.19; 95% CI: 0.10–0.35; P < 0.00001], the IT sufentanil group had a significantly higher incidence of pruritus (RR: 6.18; 95% CI: 1.18–32.46; *P* = 0.03) The IT meperidine group had a significantly lower incidence of intraoperative discomfort (2.7% vs. 13.6%; RR: 0.22; 95% CI: 0.09–0.55; *P* = 0.001), but there was a significant increase in nausea and vomiting (IT meperidine group vs. Control group: 42.7% vs. 19.4%; RR: 2.56; 95% CI: 1.14–5.75; *P* = 0.02). Meta-regression analysis based on the opioid dose and quality of the study did not impact the final inference of our result.

**Conclusion:**

IT fentanyl significantly decreased the incidence of shivering in women undergoing CD under spinal anesthesia without increasing maternal adverse events, confirming that routine use in this patient population is a good choice. IT sufentanil did not decrease the incidence of shivering. IT meperidine decreased the incidence and severity of shivering, but its use was also associated with significant nausea and vomiting.

## Background

Cesarean delivery is one of the most common operations performed. It is routinely carried out under spinal anesthesia using a combination of local anesthetics and opioids. Intrathecal (IT) addition of a lipophilic opioid to local anesthetic reduces the dose of local anesthetic, shortens the onset of block, markedly improves the quality of anesthesia, prolongs the duration of analgesia and also decreases the incidence of shivering [1].

Up to 85% of patients undergoing cesarean delivery may experience shivering after spinal anaesthesia [2, 3]. The etiology of shivering likely involves multiple mechanisms. Pregnant patients have high circulating concentrations of progesterone which may account for decreased shivering thresholds. The sympathetic blockade associated with spinal anesthesia may impair the thermoregulation causing peripheral vasodilatation. This causes the transfer of heat towards the periphery from core and enhances the heat loss through the skin. In addition, at the central nervous system level, there is increased sweating thresholds and decreased vasoconstriction [4]. The oxygen consumption (upto 600%), carbondioxide production and blood pressure may increase with shivering leading to serious hemodynamic effects in patients with compromised cardiopulmonary function. Shivering may also interfere with non-invasive patient monitoring, disrupting care in the perioperative period [2]. Thus, prevention or treatment of shivering is an important clinical goal.

Common treatment regimens for shivering include increasing the body temperature, physical warming, increasing the operating room temperature, and using various medications such as clonidine, meperidine, fentanyl and morphine [5, 6]. Sufentanil, fentanyl and meperidine, in decreasing order of lipid solubility, are used as adjuvants for spinal anesthesia in patients undergoing cesarean delivery. Several randomized controlled studies investigated the effect of these opioids on the incidence of the shivering. However, inconsistencies in the results impeded meaningful conclusions. Therefore, we performed this systematic review and network meta-analysis (SRNMA) to evaluate the effects of the multiple lipophilic neuraxial opioids on the incidence of shivering in women having cesarean delivery under spinal anesthesia.

## Methods

This meta-analysis was planned in accordance with the PRISMA-NMA guidelines (Preferred Reporting Items for Systematic Reviews for Network Meta-Analysis).

### Study selection criteria

A systematic search was performed for full reports of randomized controlled trials (RCT) that reported on the incidence of shivering in patients undergoing cesarean delivery under spinal anesthesia with IT lipophilic opioids, such as fentanyl, sufentanil and meperidine. Relevant trials had to report the incidence of shivering in both the intervention and control groups. Any studies without data on control group were excluded from the analysis. The spinal anesthetic technique should have been standardized for both the treatment and control groups and should have included the administration of IT lipophilic opioids.

### Literature search

The following databases were systematically searched for relevant studies in English language by an expert librarian: PubMed, Medline, Embase, Cochrane Central Register of Controlled Trials, Web of Science, Scopus and CINAHL. The search was conducted from 1946 to October 2019. Additional studies were identified from the reference list of retrieved reports. MeSH keywords used in the search were “prevention”, “incidence”, “shivering”, “severity”, “intrathecal”, “spinal”, “neuraxial”, “fentanyl”, “sufentanil”, “meperidine”, “lipophilic opioids”, “obstetric patients”, “parturients”, “caesarian section”, “cesarean delivery.” Data from abstracts, letters, retrospective trials, case reports and unpublished data were not considered and were excluded from the analysis.

### Study retrieval

Two investigators (YS and KK) independently reviewed the search results in a stepwise manner. Relevant studies were first selected by title review of the search results. Abstracts of the selected studies were screened to determine if the inclusion/exclusion criteria were fulfilled. Then, the full text of the selected manuscript was considered and pertinent information was collected. In case of discrepancies, a senior author (SS) was consulted to resolve the issues.

### Data collection

A data collection form was used to collect the following data: (i) study ID; (ii) (ii) drug and dose of IT opioid [fentanyl, sufentanil and meperidine]; (iii) therapeutic allocation and sample size in each group; (iv) primary outcome: outcome measures including the incidence of shivering; (v) secondary outcomes: incidence of side effects such as hypotension, intraoperative discomfort, pruritus, nausea and vomiting.

### Study quality assessment

The articles meeting the inclusion criteria were scored independently by two authors (YS and KK) for methodological quality, based on the modified Oxford score to determine the various risks of bias [7]. The key domains assessed were (1) randomization; (2) concealment of allocation; (3) double blinding; (4) flow of patients.

### Statistical analysis

#### Network Meta-analysis (NMA)

We conducted a network meta-analysis to permit comparison of the effect of multiple intrathecal lipophilic opioids across a network of trials within the same or very similar patient population: i.e. direct and indirect data were combined to try to estimate the most effective opioid to prevent the incidence of shivering [8]. Analyses were undertaken using Bayesian random–effects models via Monte Carlo Markov Chain (MCMC) simulations with non-informative prior distributions. Analyses were performed using the R studio version 1.0.153 – Inc.

Crude data (dichotomous data) were extracted from the individual studies and summarized as odds ratios (OR) with 95% credible interval (CrI). The data on the side effects were summarized as the risk ratio (RR) with 95% confidence interval. The data for the individual groups was collected and then pooled across groups using Bayesian random-effects modeling. Continuity correction was done for those cells which had zero as the outcome. Two tailed *P* < 0.05 was considered statistically significant.

For the direct data, the meta-regression and sensitivity analysis of the various subgroups was done to measure the impact of the various doses of IT opioids and quality of the studies on the incidence of shivering. Heterogeneity across studies was investigated for each group by chi-square test and calculating I^2^ to estimate the percentage of variation in study estimates that is due to heterogeneity rather than sampling error.

#### Quality of evidence in the network meta-analysis

The level of confidence in each intrathecal opioid effect, estimated by the network meta-analysis, was assessed using the CINeMA frame work [9] and GRADE approach [10]. The equality of evidence in each opioid effect was assessed for study limitation, indirectness, imprecision, inconsistency (heterogeneity and incoherence) and publication bias. Overall, certainty of the evidence was assessed using the GRADE approach. The study protocol is included in the supplementary file S1.

## Results

**Fig. 1:**
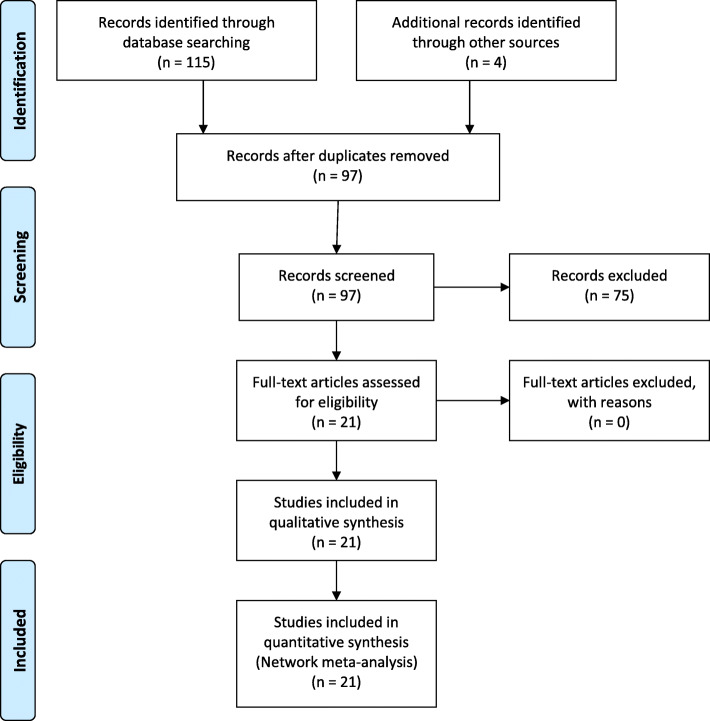
Flowchart on the literature search

Twenty-one potentially relevant articles were identified from 115 citations. Twenty-one studies consisting of 1433 patients (Control group: 590 patients in twenty-one studies; Fentanyl group:199 patients in seven studies [11–17]; Sufentanil group: 156 patients in five studies [18–22]; Meperidine group: 488 patients in ten studies [15, 23–31]) contained data regarding the effect of IT opioids on shivering (Flow chart: Fig. 1). One included study by Han et al. 2007 investigated the effect of both, fentanyl and meperidine [15]. Tables 1 and 2 summarise the systematic review of the effects of IT fentanyl, sufentanil and meperidine on shivering in women undergoing cesarean delivery. Out of twenty-one studies, ten studies investigated shivering as the primary outcome [14–16, 21, 24–26, 28–30]. Methodological validity scores determined by modified Oxford score ranged from 3 to 7.

**Table 1:** Effect of lipophilic opioids on incidence of shivering in women undergoing cesarean delivery after spinal anesthesia: A systematic review of randomized control trials presented in a tabular column

Serial No.	Study reference Study ID year [Country of origin] [Modified Oxford score-R/C/D/F]	Groups Drug & Dosage [Intrathecal administration]	Results
1	[11]Palmer et al. 1995 [USA] [2/0/2/0]	Control Vs. Fentanyl (F) 15 μg	**Group F (14) vs. Control (14)** •Incidence: 0% vs. 14.28% •Severity (Grades 3 and 4): NA **Side Effects:** •Hypotension: 0 vs. 0 •Pruritus: 7.14% vs. 28.57% •Nausea and vomiting: 50% vs 92.85% •Intraoperative discomfort: NA •Respiratory depression: 0 vs. 0
2	[20]Chen et al. 2010 [Germany] [2/0/2/2]	Control Vs. Sufentanil (S) 5 μg	**Group S (32) vs. Control (32)** •Incidence: 0% vs. 38% •Severity (Grades 3 and 4): NA **Side Effects:** •Hypotension: 19% vs. 38% •Pruritus: 31% vs. 0% •Nausea and vomiting: 31% vs. 52% •Intraoperative discomfort: NA •Respiratory depression: 0 vs. 0
3	[22]Abdollahpour et al. 2015 [Iran] [2/0/2/2]	Control Vs. Sufentanil (S) 1.5 μg	**Group S (25) vs. Control (25)** •Incidence: 48% vs. 40% •Severity (Grades 3 and 4): NA **Side Effects:** •Hypotension: 64% vs 84% •Pruritus: NA •Nausea and vomiting: 64% vs. 52% •Intraoperative discomfort: NA •Respiratory depression: NA
4	[15]Han et al. 2007 [Korea] [2/0/2/0]	Control Vs. Fentanyl (F) 12.5 μg	**Group F (20) vs. Control (20)** •Incidence: 30% vs. 65% •Severity (Grades 3 and 4): - 10% vs. 35% **Side Effects:** •Hypotension: NA •Pruritus: NA •Nausea and vomiting: NA •Intraoperative discomfort: NA •Respiratory depression: NA
5	[17]Agrawal et al. 2016 [India] [2/1/0/1]	Control Vs. Fentanyl (F) 25 μg	**Group F (20) vs. Control (20)** •Incidence: 10% vs. 30% •Severity (Grades 3 and 4): NA **Side Effects:** •Hypotension: 75% vs. 75% •Pruritus: 30% vs. 0% •Nausea and vomiting: 15% vs. 70% •Intraoperative discomfort: NA •Respiratory depression: NA
6	[16]Sadegh et al. 2012 [Iran] [2/1/2/2]	Control Vs. Fentanyl (F) 25 μg	**Group F (40) vs. Control (40)** •Incidence: 10% vs. 75% •Severity (Grades 3 and 4): 0 vs. 23% **Side Effects:** •Hypotension: 75% vs. 77.5% •Pruritus: 30% vs. 0% •Nausea and vomiting: 18.95% vs. 67.5% •Intraoperative discomfort: 3% vs. 35% •Respiratory depression: 0 vs. 0
7	[19]Qian et al. 2009 [China] [2/1/0/2]	Control Vs. Sufentanil (S) 5 μg	**Group S (40) vs. Control (40)** •Incidence: 20% vs. 60% •Severity (Grades 3 and 4): NA **Side Effects:** •Hypotension: 20% vs. 55% •Pruritus: 0 vs. 0 •Nausea and vomiting: 0 vs. 0 •Intraoperative discomfort: NA •Respiratory depression: 0 vs. 0
8	[21]Locks et al. 2012 [Brazil] [2/0/0/2]	Control Vs. Sufentanil (S) 2.5 μg	**Group S (40) vs. Control (40)** •Incidence: 32.5% vs. 62.5% •Severity (Grades 3 and 4): NA **Side Effects:** •Hypotension: NA •Pruritus: NA •Nausea and vomiting: NA •Intraoperative discomfort: NA •Respiratory depression: NA
9	[14]Techanivate et al. 2005 [Thailand] [2/1/2/0]	Control Vs. Fentanyl (F) 20 μg	**Group F (30) vs. Control (30)** •Incidence: 20% vs. 50% •Severity (Grades 3 and 4): 3.33% vs. 13.33% **Side Effects:** •Hypotension: 36.7% vs. 50% •Pruritus: 66.66% vs. 40% •Nausea and vomiting: 33.33% vs. 23.33% •Intraoperative discomfort: 0 vs. 26.7% •Respiratory depression: NA
10	[13]Kang et al. 1998 [Taiwan] [2/0/0/1]	Control Vs. Fentanyl (F) 25 μg	**Group F (15) vs. Control (15)** •Incidence: 0% vs. 33.3% •Severity (Grades 3 and 4): NA **Side Effects:** •Hypotension: 20% vs. 40% •Pruritus: 93.5% vs. 0% •Nausea and vomiting: 60% vs. 66.6% •Intraoperative discomfort: 0 vs. 13.3% •Respiratory depression: 0 vs. 0
11	[12]Chu et al. 1995 [China] [2/0/0/1]	Control Vs. Fentanyl (F) 7.5 μg Fentanyl (F) 10 μg Fentanyl (F) 12.5 μg Fentanyl (F) 15 μg	**Group F 7.5 (15) vs. F 10 (15) vs. F 12.5 (15) vs. F 15 (15) vs. Control (15)** •Incidence: 66.7% vs. 46.6% vs. 33.3% vs. 26.6% vs. 66.7% •Severity (Grades 3 and 4): NA **Side Effects:** •Hypotension: 26.6% vs. 40% vs. 26.6% vs. 26.6% vs. 33.3% •Pruritus: 20% vs. 26.6% vs. 40% vs. 53.3% vs. 0% •Nausea and vomiting: 53.3% vs. 53.3% vs. 46.6% vs. 46.6% vs. 46.6% •Intraoperative discomfort: 41.2% vs. 20% vs. 0% vs. 0% vs. 66.7% •Respiratory depression: 0
12	[18]Lin et al. 1998 [China] [2/0/2/0]	Control Vs. Sufentanil (S) 10 μg	**Group S (19) vs. Control (22)** •Incidence: 21% vs. 4.5% (4 vs. 1) •Severity (Grades 3 and 4): 0 vs. 4.5% (1) **Side Effects:** •Hypotension: 89.47% vs. 57.89% (17 vs. 11) •Pruritus: 42.1% vs. 4.5% (8 vs. 1) •Nausea and vomiting: 31.57% vs. 57.89% (6 vs. 11) •Intraoperative discomfort: 36.84% vs. 68.42% (7 vs. 13) •Respiratory depression: NA

R/C/D/F: Randomization (2)/Concealment of allocation (1)/Double blinding (2)/Flow of patients (2); NA: Not Available

Modified Oxford Score varies from 0 to 7

**Table 2:** Effect of Meperidine on incidence of shivering in women undergoing cesarean delivery after spinal anesthesia: A systematic review of randomized control trials presented in a tabular column

Serial No.	Study Reference Study ID year [Country of origin] [Modified Oxford score-R/C/D/F]	Groups Drug, dosage (Intrathecal Administration)	Results
1	[23]Yu et al. 2002 [China] [2/1/2/2]	Control Vs. Meperidine (M) 10 mg	**Group M (20) vs. Control (20)** •Incidence: 15% vs. 40% •Severity (Grades 3 and 4): NA **Side Effects:** •Hypotension: 70% vs. 55% •Pruritus: 0 vs. 0 •Nausea and vomiting: 55% vs. 15% •Intraoperative discomfort: 0% vs. 10% •Respiratory depression: 0 vs. 0
2	[28]Khan et al. 2011 [Iran] [2/0/2/2]	Control Vs. Meperidine (M1) 12.5 mg Meperidine (M2) 25 mg	**Group M1 (24) vs. M2 (24) vs. Control (24)** •Incidence: 20.83% vs. 4.16% vs. 58.33% •Severity (Grades 3 and 4): 0 vs. 0 vs. 16.66% **Side Effects:** •Hypotension: 50% vs. 45.8% vs. 41.7% •Pruritus: 0 vs. 0 vs. 0 •Nausea and vomiting: 25% vs. 75% vs. 4.2% •Intraoperative discomfort: NA •Respiratory depression: 0 vs. 0 vs. 0
3	[31]Atalay et al. 2010 [Turkey] [2/0/2/2]	Control Vs. Meperidine (M1) 25 mg Meperidine (M2) 30 mg Meperidine (M3) 35 mg	**Group M1 (20) vs. M2 (20) vs. M3 (20) Control (20)** •Incidence: 0% vs. 0% vs. 0% vs. 50% •Severity (Grades 3 and 4): NA **Side Effects:** •Hypotension: 20% vs. 30% vs. 55% vs. 65% •Pruritus: 10% vs. 35% vs. 45% vs 0 •Nausea and vomiting: 25% vs. 45% vs. 75% vs. 75% •Intraoperative discomfort: 0 vs. 0 vs. 0 vs. 0 •Respiratory depression: 0 vs. 0 vs. 0 vs. 0
4	[15]Han et al. 2007 [Korea] [2/0/2/0]	Control Vs. Meperidine (M) 12.5 mg	**Group M (20) vs. Control (20)** •Incidence: 20% vs. 65% •Severity (Grades 3 and 4): 5% vs. 35% **Side Effects: NA**
5	[26]Hong et al. 2005 [South Korea] [2/1/2/2]	Control Vs. Meperidine (M) 10 mg	**Group M (30) vs. Control (30)** •Incidence: 3.3% vs. 23.3% •Severity (Grades 3 and 4): 0% vs. 20% **Side Effects: NA**
6	[24]Denis Roy et al. 2004 [Canada] [2/0/2/0]	Control Vs. Meperidine (M) 0.2 mg/kg (15 mg average)	**Group M (20) vs. Control (20)** •Incidence: 45% vs. 85% •Severity (Grades 3 and 4): 10% vs. 45% **Side Effects:** •Hypotension: NA •Pruritus: 0 vs. 0 •Nausea and vomiting: 0 vs. 0 •Intraoperative discomfort: NA •Respiratory depression: 0 vs. 0
7	[29]Rastegarian et al. 2013 [Iran] [2/1/2/2]	Control Vs. Meperidine (M) 0.2 mg/kg (15 mg average)	**Group M (50) vs. Control 50)** •Incidence: 8% vs. 28% •Severity (Grades 3 and 4): 0% vs. 18% **Side Effects:** •Hypotension: 14% vs 12% •Pruritus: 0 vs. 0 •Nausea and vomiting: 18% vs. 0 •Intraoperative discomfort: NA •Respiratory depression: 0 vs. 0
8	[25]Anaraki et al. 2012 [Iran] [2/1/2/2]	Control Vs. Meperidine (M1) 0.2 mg/kg (15 mg average) Meperidine (M2) 0.3 mg/kg (25 mg average) Meperidine (M3) 0.4 mg/kg (30 mg average)	**Group M1 (38) vs. M2 (38) vs. M3 (39) Control (38)** •Incidence: 37.5% vs. 27.5% vs. 15% vs. 47.5% •Severity (Grades 3 and 4): 17.5% vs. 7.5% vs. 2.5% vs. 30% **Side Effects:** •Hypotension: NA •Pruritus: 28.21% vs. 38.46% vs. 48.72% vs. 25.64% •Nausea and vomiting: 15.4% vs. 25.9% vs. 35.8% vs. 8% •Intraoperative discomfort: 4.6% vs. 4.8% vs. 4.3% vs. 17.6% •Respiratory depression: 0 vs. 0 vs. 0 vs. 0
9	[27]Imarengiaye et al. 2011 [Nigeria] [2/1/2/1]	Control Vs. Meperidine (M) 7.5 mg	**Group M (25) vs. Control (25)** •Incidence: 0% vs. 4% •Severity (Grades 3 and 4): NA **Side Effects:** •Hypotension: 40% vs. 8% •Pruritus: 0 vs. 0 •Nausea and vomiting: 20% vs. 0% •Intraoperative discomfort: 0% vs. 16% •Respiratory depression: 0 vs. 0
10	[30]Shami et al. 2016 [Iran] [2/1/2/2]	Control Vs. Meperidine (M) 5 mg Meperidine (M) 10 mg	**Group M5 (50) vs. Group M10 (50) vs. Control (50)** •Incidence: 13 vs. 3 vs. 25 (26% vs. 6% vs. 3%) •Severity (Grades 3 and 4): 0 vs 0 vs. 1 (2%) **Side Effects:** •Hypotension: 33 vs. 37 vs. 34 (66% vs. 74% vs. 68%) •Pruritus: 3 vs. 13 vs. 0 (6% vs. 26% vs. 0) •Nausea and vomiting: 38 vs. 40 vs. 25 (76% vs. 80% vs. 50%) •Intraoperative discomfort: NA •Respiratory depression: NA

R/C/D/F: Randomization (2)/Concealment of allocation (1)/Double blinding (2)/Flow of patients (2); NA: Not Available

Modified Oxford Score varies from 0 to 7

### Network meta-analysis (NMA)

The twenty-one studies included in this network meta-analysis investigated the effect of three interventions: fentanyl (seven studies [11–17]), sufentanil (five studies [18–22]), and meperidine (ten studies [15, 23–31]), with four comparison groups. Six pairwise comparisons and four direct comparisons were conducted. Table 3 summarises the data on the effect of intrathecal opioids on the incidence of shivering. Out of the twenty-one studies comprising 1433 patients, 199 patients received fentanyl [11–17], 156 patients received sufentanil [18–22], 488 patients received meperidine [15, 23–31] and 590 patients were in the control group.

**Table 3:** Network meta-analysis: Estimates of direct effect, indirect effect and mixed effect with quality ratings according to GRADE approach, for the incidence of shivering in women undergoing caesarean delivery with intrathecal lipophilic opioids

	Direct evidence		Indirect evidence		Mixed Evidence Bayesian Network meta-analysis	
Comparison	OR (95% CI)	Quality of evidence	OR (95% CI)	Quality of evidence	OR (95% CrI)	Quality of evidence
^e^Meperidine vs. Control	0.17 (0.09–0.31)	Moderate	0.05 (0.00–3.16)	Moderate	0.12 (0.05–0.29)	Moderate^a^
^e^Fentanyl vs. Control	0.16 (0.07–0.35)	Low	0.41 (0.01–16.17)	Low	0.13 (0.04–0.35)	Low^ba^
Meperidine vs. Suphentanil	–	–	0.40 (0.14–1.13)	Low	–	Low^c^
Fentanyl vs. Suphentanil	–	–	0.41 (0.13–1.27)	Low	–	Low^ac^
Suphentanil vs. Control	0.42 (0.18–0.98)	Low	–		–	Low^d^
Fentanyl vs. Meperidine	1.71 (0.25–11.73)	Low	0.87 (0.30–2.50)	Low	1.02 (0.40–2.57)	Low^ac^

OR: Odds ratio; CI: Confidence Interval; CrI: Credible Interval; ^a^: rated down for Indirectness; ^b^: Contributing direct evidence of low quality; ^c^: rated down for major concern in Imprecision; ^d^: rated down for Heterogeneity; ^e^: Statistically significant results

**Fig. 2:**
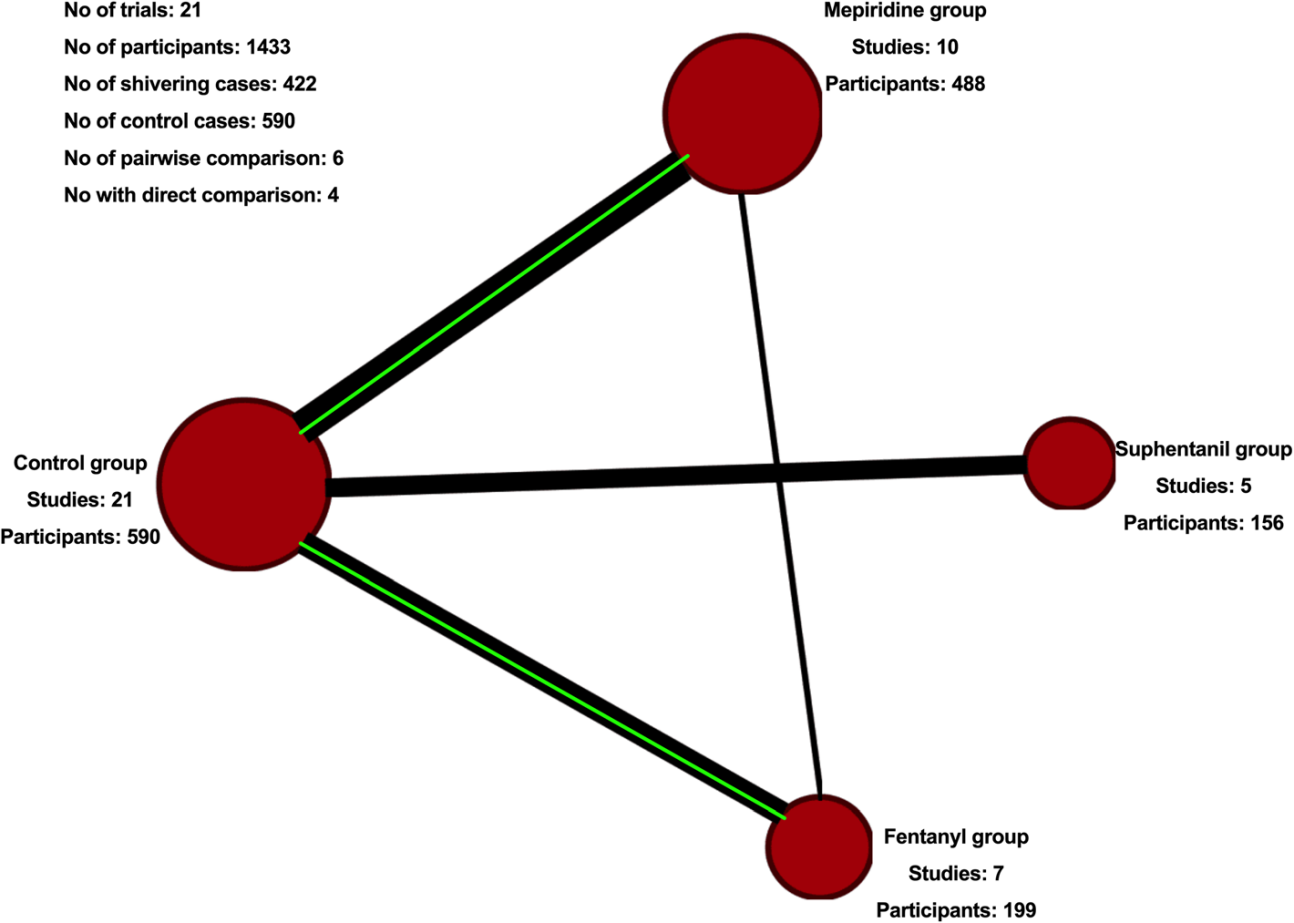
Network diagram comparing the various classes of drugs. Evidence network of randomized controlled trials comparing the effects of drugs to prevent shivering in women undergoing cesarean delivery with intrathecal lipophilic opioids. The size of the circle is proportional to the number of participants randomized to that treatment. Width of the lines is proportional to the number of trials for that comparison. The green line indicates the statistically significant results between the compared groups

Table 3 provides the effect estimates of direct, indirect and mixed network meta-analysis with quality of evidence rating according to the GRADE approach. Figure 2 displays the network diagram comparing the various intrathecal lipophilic opioids to prevent incidence of shivering in women undergoing cesarean delivery. Supplementary file S2 and S3 show the contribution matrix and league table for comparison of all classes of drugs.

### Fentanyl

Data on the incidence of shivering with IT fentanyl (7 RCTs, *n* = 199 patients) were available in all the studies [11–17]. The mixed evidence from the network meta-analysis showed that the incidence of shivering was significantly lower in the IT fentanyl group compared to the control group [IT Fentanyl vs. Control: 22.11% vs. 51.94%; Pooled Odds Ratio (OR): 0.13; 95% Credible Interval (CrI): 0.04 to 0.35; *P* = 0.0004]. The funnel plot and influential analysis on the direct data identified Sadegh et al.2003 as the outlier and contributed the maximum heterogeneity to the end estimate. When this study was excluded and summary estimates were recalculated, the end estimate increased to 0.51(0.36 to 0.71); *P* < 0.0001 and heterogeneity decreased to zero (not shown in the figure). The Begg’s test (*P* = 0.089) and Egger regression test (*P* = 0.2077) did not show any evidence of publication bias. Fail-safe N test showed 113 studies required to increase the *p* value to more than alpha (> 0.05), indicating the absence of publication bias (not shown in the figure). Fentanyl was administered in the dose range of 7.5 to 25 microgram and there was no difference in the outcomes across this dose range (Coefficient − 0.043; 95% CI: − 0.0963 to 0.0103; *P* = 0.1139).

### Sufentanil

Data on the incidence of shivering with IT sufentanil (5 RCTs, *n* = 156 patients) were available in all the studies [18–22]. The mixed evidence from the network meta-analysis showed that the incidence of the shivering was not significantly lower with IT sufentanil when compared to the control group. (IT Sufentanil vs. Control: 23.71% vs. 45.28%; OR: 0.37; 95% CrI: 0.11 to 1.22; *P* = 0.23). Meta-regression analysis based on the IT sufentanil dose did not change the final inference of the result (Coefficient 0.0919; 95% CI: − 0.2495 to 0.4333; *P* = 0.5977).

### Meperidine

Data on the incidence of shivering with IT meperidine were available in all the 10 studies [15, 23–31]. The mixed evidence from the network meta-analysis showed that the incidence of shivering was lower in the meperidine group compared to the control group (IT Meperidine vs. Control: 15% vs. 44.2%; OR: 0.12; 95% CrI: 0.05 to 0.29; *P* < 0.00001). For the direct data, the Begg’s test (*P* = 0.7544) and Egger regression test (*P* = 0.1628) did not show any evidence of publication bias. Fail-safe N test showed 85 studies required to increase the *p* value to more than alpha (> 0.05), indicating the absence of publication bias. Meperidine was used in the dose range of 5–35 mg and there was no difference in the outcomes across this dose range (Coefficient − 0.0215; 95% CI: − 0.0649 to 0.0219; *P* = 0.3314). Meta-regression and sensitivity analysis based on the quality of the study for the various subgroups slightly changed the end estimate, but did not change the final inference of our results (Table 4).

**Table 4:** Study Quality assessment: Meta-regression and sensitivity analysis

Subgroups	Quality of study (No. of studies)	Point Estimate	95% CI	I^**2**^	Meta-Regression	
					Coefficient (Standard Error)	***p***-value
Fentanyl	Good (2) Poor - moderate (5)	0.24 0.50	0.08–0.72 0.32–0.79	69% 13%	−0.507 (0.393)	0.1969
Sufentanil	Good (3) Poor - moderate (2)	0.40 1.23	0.1–1.66 0.14–10.7	85% 76%	−0.980 (1.113)	0.378
Low dose Meperidine	Good (7) Poor - moderate (2)	0.41 0.46	0.27–0.61 0.28–0.75	26% 8%	−0.125 (0.376)	0.7391
High dose Meperidine	Good (3) Poor - moderate (0)	0.10 -	0.01–1.0 -	81% -	–	–

CI: Confidence Interval. Study quality scores were obtained from the modified oxford scoring system. Study was considered good when assigned score was equal or greater than 5 out of 7. *P*-values are based on random-effects model

### Side effects

#### IT fentanyl

The IT fentanyl group had a significantly lower incidence of intraoperative discomfort (IT Fentanyl vs. Control: 6.89% vs. 34%; Risk Ratio (RR): 0.19; 95% CI: 0.10–0.35; *P* < 0.00001), but there was no significant difference in other maternal adverse events like pruritus (IT Fentanyl vs. Control: 38.14% vs. 18.79%; RR: 2.03; 95% CI: 0.82–5.05; *P* = 0.13), nausea and vomiting (IT Fentanyl vs. Control: 39.10% vs. 58.20%; RR: 0.66; 95% CI: 0.42–1.05; *P* = 0.08) and hypotension (IT Fentanyl vs. Control: 43.57% vs. 54.47%; RR: 0.93; 95% CI: 0.78–1.12; *P* = 0.45).

#### IT Sufentanil

The IT sufentanil group had a significantly higher incidence of pruritus (IT Sufentanil vs. Control: 20.87% vs. 2.12%; RR: 6.18; 95% CI: 1.18–32.46; *P* = 0.03), but there was no significant difference in other maternal adverse events like hypotension (IT Sufentanil vs. Control: 40.51% vs. 55.46%; RR: 0.74; 95% CI: 0.37–1.47; *P* = 0.39), nausea and vomiting (IT Sufentanil vs. Control: 28.44% vs. 35.29%; RR: 0.83; 95% CI: 0.53–1.29; *P* = 0.40). IT sufentanil did not significantly decrease the intraoperative discomfort compared to the control group (IT Sufentanil vs. Control: 36.84% vs. 59.09%; RR: 0.62; 95% CI: 0.31–1.24; *P* = 0.18).

#### IT Meperidine

The IT Meperidine group had significantly lower incidence of intraoperative discomfort (IT Meperidine vs. Control: 2.7% vs. 13.6%; RR: 0.22; 95% CI: 0.09–0.55; *P* = 0.001). There was a significant increase in nausea and vomiting (IT Meperidine vs. Control: 42.7% vs. 19.4%; RR: 2.56; 95% CI: 1.14–5.75; *P* = 0.02), but there was no significant difference in other maternal adverse events between the two groups, like hypotension (IT Meperidine vs. Control: 46.9% vs. 41.8%; RR: 0.96; 95% CI: 0.67–1.37; *P* = 0.82) and pruritus (IT Meperidine vs. Control: 18.9% vs. 6%; RR: 0.63; 95% CI: 0.82–3.24; *P* = 0.17).

### Quality of the evidence in network estimates

Supplementary files S4 and S5 show the rankogram and various domains examined to assess the quality of evidence in the network meta-analysis. Most of the included studies in the network meta-analysis were randomized double blind controlled studies with no, or some, concerns in the study limitation. To assess the imprecision, effect estimates of the relative treatments lower than 0.95 and greater than 1.05 were considered to be clinically significant. The data were collected from different studies, across different countries, at varying time intervals and the network model showed some degree of incoherence (χ^2^ statistics: 0.336; d(f): 2; *p* value: 0.846). The estimated value of between-study variance for the network meta-analysis is 0.412 indicating some heterogeneity and consistency in the network model. Overall, some of the comparisons were rated down for imprecision, heterogeneity and incoherence (inconsistency), thus the quality of the evidence for the effect estimates was low according to the GRADE approach.

## Discussion

In this systematic review evaluating the effects of lipophilic opioids to prevent or reduce shivering in patients having spinal anesthesia for cesarean delivery, fentanyl was found to be more effective than sufentanil and meperidine, however, there was no significant difference between direct or indirect comparison between fentanyl and meperidine. IT fentanyl (7.5–25 mcg) was found to decrease the incidence and severity of shivering as well as to improve the quality of spinal anesthesia in women having CD [32]. Fentanyl is a highly ionized, lipophilic μ-receptor agonist. When it is administered intrathecally, the unionized component is rapidly transferred into the spinal cord. IT fentanyl used with bupivacaine, in doses of 15 microgram has been shown to be effective in prolonging the duration of analgesia, and it also exerts an anti-nausea effect in such small doses. This is probably due to decreased nociceptive stimulation from peritoneal manipulation and uterine exteriorization due to augmented quality of spinal block caused by fentanyl [11]. The reduction of shivering may be attributable to the effect of fentanyl that was added into the subarachnoid space on the thermo-regulator and spinal affect afferent thermal inputs at the spinal cord [33]. It is shown that fentanyl can reduce the intensity and severity of shivering up to 3 h after spinal anesthesia, including the time before delivery of the baby. This reduces the requirement of intravenous medications to treat shivering before delivery, thereby decreasing any harmful effects of medications on the baby [14]. The main detriment of preventing shivering is the fall in body temperature as shivering is a protective autonomic response against hypothermia. However, fentanyl lowered the core temperature for only 2 h, with return to baseline temperature in the third hour, without any harmful effects on the patient [14].

We found that addition of IT fentanyl was associated with lowest incidence of intraoperative discomfort due to increase in the quality of analgesia. The incidence of pruritus with the administration of opioid into the subarachnoid space was reported to be 67% for fentanyl, and 80% for sufentanil [34]. But, several studies have shown that there was no increase in the incidence of pruritus with IT fentanyl doses less than 50 microgram [35–37]. Also, the pruritus associated with IT fentanyl and sufentanil is transient and resolves rapidly, usually without any need for treatment [11, 21]. The results of our study are supported by similar findings, which compared nine different neuroaxial adjuvants and found fentanyl to be the optimal choice [38].

Sufentanil is highly lipophilic with a higher affinity to opioid receptors, with a less cephalad spread and a much higher analgesic potency when compared with fentanyl or morphine [39–41]. It is well accepted for use in spinal anesthesia together with local anesthetics for caesarean delivery [20]. The combination of hyperbaric ropivacaine 10 mg with sufentanil 5 μg produced effective spinal anaesthesia for caesarean delivery with significantly less hypotension, vomiting and shivering, shorter duration of motor blockade and longer lasting analgesia than hyperbaric ropivacaine15 mg [19]. Our results show, however, that IT sufentanil was not effective in decreasing and severity of shivering in patients undergoing CD. While this finding could be due to the fact that this meta-analysis was limited by the small number of available studies evaluating this outcome, a previous meta-analysis on IT and epidural sufentanil had similar findings, and the authors postulated that the low dose range of IT and epidural sufentanil used (1.5–20 μg) may not be effective in decreasing shivering [42, 43].

Meperidine is an opioid with intermediate lipid solubility and is unique in having local anesthetic properties [44]. It has been used as a sole agent for spinal anesthesia for cesarean delivery [45–47]. It prolongs postoperative analgesia for up to 4 h, which is considerably lower compared to IT morphine, which can provide analgesic effect for up to 24 h [48–50]. An incidence of pruritus of 10.7–32% has been reported with the use of ≥50 mg of IT meperidine, which is much lower than IT morphine [33, 47, 51]. Meperidine is shown to be effective against shivering. While the mechanism of action is not fully understood, the anti-shivering effect of intravenous meperidine is due to its effect on the kappa opioid receptor and decreases the threshold of shivering [26, 52–55]. Possible suggested mechanisms for the anti-shivering effect of meperidine include k-opioid receptor activity, anticholinergic action, biogenic monoamine reuptake inhibition, NMDA receptor antagonism, or stimulation of alpha 2–adrenoceptors, and possibly modulating the heat loss caused by vasodilatation after spinal anesthesia [54, 56–58]. Meperidine slightly increases the threshold for sweating, significantly decreases the threshold for vasoconstriction, and reduces the threshold for shivering [59]. We found that IT meperidine was associated with nausea and vomiting, which limits its clinical efficacy.

### Limitations

The doses of IT fentanyl, sufentanil and meperidine differed among the studies, which is a limitation of this systematic review, although the meta-regression ruled out the effect of the varying doses on our primary outcome. Overall, this systematic review and meta-analysis provides the best summary of the effect of the IT lipophilic opioids on shivering after cesarean delivery.

## Conclusion

IT fentanyl significantly decreased the incidence and severity of shivering in women undergoing cesarean delivery under spinal anesthesia without increasing maternal adverse events, indicating that its routine use in this patient population should be considered. IT sufentanil did not decrease the incidence or severity of shivering. IT meperidine also decreased the incidence and severity of shivering, but it was associated with significant nausea and vomiting.

## Supplementary information

**Additional file 1: Supplementary file S1.**Study protocol. **Supplementary file S2.** Contribution matrix. **Supplementary file S3.** League table. **Supplementary file S4.** Rankogram with SUCRA. **Supplementary file S5.** Quality of evidence in the network meta-analysis assessed in the following domains: study limitation, imprecision, heterogeneity, incoherence, indirectness, and final report.

## Data Availability

We will submit the data in the journal “Data in Brief” under the title “Data for the incidence of shivering in patients undergoing caesarean section under spinal anesthesia with intrathecal lipophilic opioids. A bayesian network meta-analysis data set”.

## Abbreviations

BP: Blood Pressure
CD: Caesarean delivery
CrI: Credible Intervals
IT: Intrathecal
MCMC: Monte Carlo Markov Chain
NMA: Network Meta-analysis
OR: Odds Ratio
PRISMA-NMA: Preferred Reporting Items for Systematic Reviews for Network Meta-Analysis.
RCT: Randomized Controlled Trials
SRNMA: systematic review and network meta-analysis
