# Supplementary material for: Effect of intrathecal lipophilic opioids on the incidence of shivering in women undergoing cesarean delivery after spinal anesthesia: a systematic review and bayesian network meta- analysis of randomized controlled trials
Source: BMC Anesthesiol. 2020 Aug 26;20:214. doi: 10.1186/s12871-020-01116-5 (PMC7448354; doi:10.1186/s12871-020-01116-5)
Supplement: Supplementary file 1 — Additional file 1: Supplementary file S1.Study protocol. Supplementary file S2. Contribution matrix. Supplementary file S3. League table. Supplementary file S4. Rankogram with SUCRA. Supplementary file S5. Quality of evidence in the network meta-analysis assessed in the following domains: study limitation, imprecision, heterogeneity, incoherence, indirectness, and final report. [file 12871_2020_1116_MOESM1_ESM.pdf]

S1 Protocol Study protocol for systematic review and network meta-analysis of the "Effect of Intrathecal Lipophilic Opioids on the Incidence of Shivering in Women Undergoing Cesarean Delivery after Spinal Anesthesia: A Systematic Review and Network Meta-analysis of Randomized Controlled Trials"

Objective:

In this systematic review and network meta-analysis, we aim to analyse the efficacy of intrathecal lipophilic opioids on the incidence of shivering in women undergoing cesarean delivery after spinal anesthesia.

Inclusion criteria:

Study type:

- All randomized controlled trials were eligible to enter network meta-analysis.
- We will include all studies which were published as original reports and present data on the incidence of shivering with intrathecal lipophilic opioids.

Participants:

- Adult women (>18 years) undergoing caesarean section under spinal anesthesia with intrathecal lipophilic opioids will be included.

Definition of exposition:

- All studies that used the intrathecal lipophilic opioids like fentanyl, sufentanil and meperidine for cesarean delivery under spinal anesthesia were included

Outcome variable:

- All studies which reported on the incidence of shivering in patients undergoing cesarean delivery under spinal anesthesia with intrathecal lipophilic opioids will be included.

Outcome measures:

- The OR will either be extracted from the published article or calculated by the authors.

- If the OR is not directly reported or cannot be readily extracted from the published data, the reviewers will contact the corresponding authors for additional information (e.g., data provided in 2x2 contingency tables).

Publication type:

- Full published papers excluding case reports, review articles, and editorials will be eligible.

Search Methods:

We will search the following electronic databases:

- MedLine (via PubMed)
- EMBASE
- Scopus
- Web of Science
- Google Scholar
- CINAHL
- Cochrane Central Register of Controlled Trial
- We will search literature from 1946 upto July 2019 for full reports of randomized controlled trials (RCT) in English language that present information on the incidence of shivering in patients undergoing cesarean delivery under spinal anesthesia with intrathecal lipophilic opioids such as fentanyl, sufentanil and meperidine.

The following keywords will be employed:

The search included the combination of the following MESH key words: “prevention”, “incidence”, “severity”, “fentanyl”, “sufentanil”, “meperidine”, “pethidine”, “intrathecal”, “spinal”, “neuraxial”, “shivering”, “obstetric patients”, “parturients”, “caesarian section”, “cesarean delivery.”

Additionally, bibliographies of identified publications and published reviews will be hand searched for potentially relevant articles. Authors will be contacted if data, methods and/or parameter definitions provided from the respective studies are unclear.

Reviews:

All references cited in the identified reviews will be manually searched for potentially relevant studies.

Data collection:

Two reviewers (YS, KK) will independently scrutinize the list of titles, and if available the abstracts, to determine potential usefulness of the article. Final selection will be based on the full text of potentially relevant articles by the two reviewers independently. In case of discrepancies, senior author (I.S) will be consulted to resolve the issues. Study quality will be measured using the Modified Oxford Score (Jadad AR et al. 1996)

The following study characteristics will be extracted: (i) study ID; (ii) country of origin; (iii) drug and dose of intrathecal opioid used; (iv) therapeutic allocation and sample size in each group; (v) outcome measures including the incidence and severity of shivering; (vi) incidence of side effects such as hypotension, intraoperative discomfort, pruritus, nausea and vomiting. From all eligible studies, relevant data will be extracted in duplicate, using a standardized data extraction sheet. An independent reviewer will confirm all data entries and will check at least twice for completeness and accuracy.

Meta-analysis:

Dichotomous comparisons:

- Dichotomous data on the incidence of shivering will be extracted and summarized using mixed effect odds ratio (OR) with 95% confidence intervals (CI) using the network meta-analysis.

- Random-effects models to estimate the pooled odds ratios for the incidence of shivering will be constructed across all studies.

Assessment of heterogeneity:

- Impact of heterogeneity will be assessed by calculating the I^2 according to Higgins et al. (Higgins JP et al. 2003).

Subgroup/Sensitivity analyses:

- To identify potential sources of heterogeneity and sources of bias, studies will be stratified by therapeutic group, study quality scores and any other confounding factors

Influence analysis

- Robustness of the pooled estimates will be checked by influence analyses. Each of the studies will be individually omitted from the data set, followed in each case by recalculation of the pooled estimate of the remaining studies.

Evaluation of bias and confounding:

Publication bias:

- Publication bias will be assessed by inspection of the funnel plot and formal testing for funnel plot asymmetry, using Begg's test (Sterne JA et al. 2001).

Discussion and Evaluation:

- The results will be critically and integratively discussed.

References:

1. Jadad AR, Moore RA, Carroll D, Jenkinson C, Reynolds DJ, Gavaghan DJ, McQuay HJ. Assessing the quality of reports of randomized clinical trials: is blinding necessary? Control Clin Trials 1996;17:1–12.
2. Higgins JP, Thompson SG, Deeks JJ, Altman DG (2003) Measuring inconsistency in meta- analysis. BMJ 327: 557-560.
3. Sterne JA, Egger M, Smith GD (2001) Systematic review in health care: investigating and dealing with publication and other biases in meta-analysis. BMJ 323: 101-105.

Supplementary file S2: Contribution Matrix; Per study contribution

	Mixed effect				Indirect effect	
random OR	Fentanyl: Meperidine	Fentanyl: Control	Meperidine: Control	Suphentanil: Control	Fentanyl: Suphentanil	Meperidine: Suphentanil
1	2.1196	4.6597	0.2105	0	2.3298	0.1403
2	8.0453	17.6865	0.799	0	8.8432	0.5326
3	2.2792	5.0105	0.2263	0	2.5052	0.1509
4	8.3249	18.3012	0.8267	0	9.1506	0.5512
5	29.7536	20.1352	11.8612	0	11.2073	6.6882
6	7.6223	16.7565	0.757	0	8.3783	0.5046
7	5.2851	11.6185	0.5248	0	5.8093	0.3499
8	0	0	0	10.4803	5.0188	5.1023
9	0	0	0	27.6772	13.2542	13.4747
10	0	0	0	7.2397	3.467	3.5246
11	0	0	0	29.4172	14.0874	14.3217
12	0	0	0	25.1856	12.061	12.2616
13	3.8409	0.6125	8.9059	0	0.4083	4.453
14	3.8651	0.6164	8.962	0	0.4109	4.481
15	6.9104	1.102	16.023	0	0.7347	8.0115
16	2.3819	0.3799	5.523	0	0.2532	2.7615
17	1.2314	0.1964	2.8553	0	0.1309	1.4276
18	5.0244	0.8012	11.65	0	0.5342	5.825
19	4.9661	0.792	11.5149	0	0.528	5.7575
20	6.8647	1.0947	15.917	0	0.7298	7.9585
21	1.4851	0.2368	3.4435	0	0.1579	1.7218

Contribution Matrix; Per comparison contribution

random OR	Fentanyl: Meperidine	Fentanyl: Control	Meperidine: Control	Suphentanil: Control
**Mixed estimates**				
Fentanyl: Meperidine	20.55	39.725	39.725	0
Fentanyl: Control	6.335	87.33	6.335	0
Meperidine: Control	3.945	3.945	92.11	0
Suphentanil: Control	0	0	0	100
**Indirect estimates**				
Fentanyl: Suphentanil	4.2233	43.665	4.2233	47.8883
Meperidine: Suphentanil	2.63	2.63	46.055	48.685

Supplementary file S3: League Table

**Fentanyl**	**1.021 (0.405 - 2.572)**	**0.410 (0.132 - 1.270)**	**0.173 (0.081 - 0.366)**
**0.980 (0.389 - 2.469)**	**Meperidine**	**0.401 (0.142 - 1.132)**	**0.169 (0.093 - 0.308)**
**2.440 (0.787 - 7.566)**	**2.491 (0.883 - 7.023)**	**Suphentanil**	**0.421 (0.181 - 0.981)**
**5.796 (2.733 - 12.291)**	**5.916 (3.249 - 10.773)**	**2.375 (1.019 - 5.533)**	**Control**

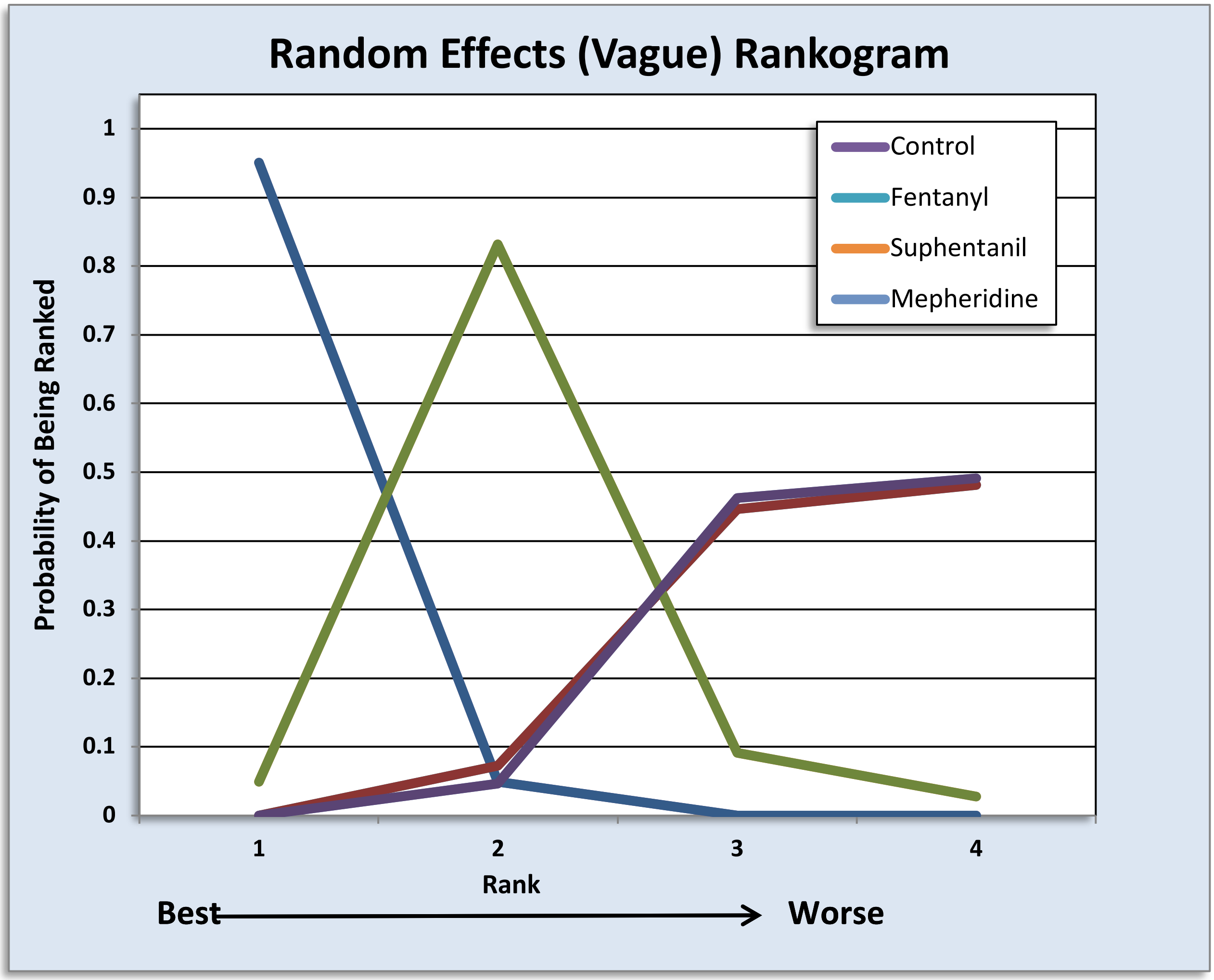

Treatment	SUCRA
**Control**	0.9836
**Suphentanil**	0.6342
**Fentanyl**	0.1971
**Meperidine**	0.1851

Within-study bias

21 total studies

21 : low

Risk of bias contributions

The bar chart shows the contributions of each piece of study to the network estimate

Save Chart

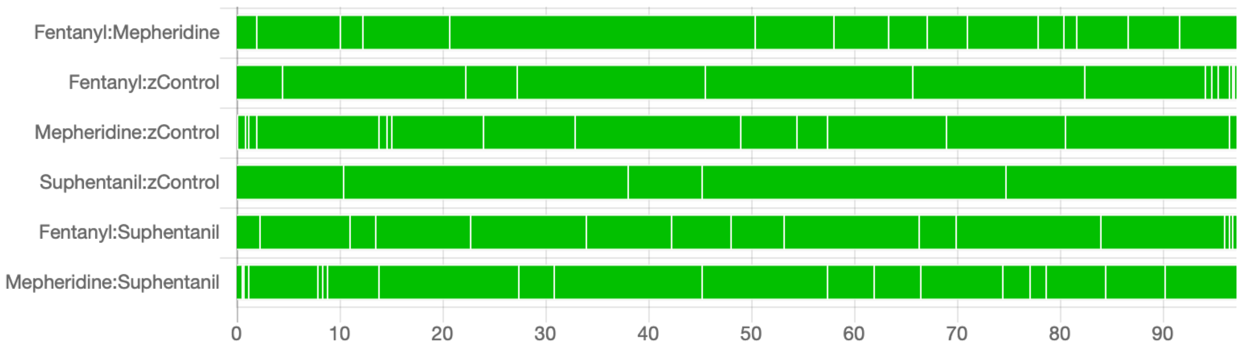

Selected rule: Average RoB

Reset Proceed

Comparison Fentanyl:Mepheridine
Evidence: mixed

Majority RoB: **No concerns**
Average RoB: **No concerns**
Highest RoB: **No concerns**
NMA judgment No concerns

Comparison Fentanyl:zControl
Evidence: mixed

Majority RoB: **No concerns**
Average RoB: **No concerns**
Highest RoB: **No concerns**
NMA judgment No concerns

Comparison Mepheridine:zControl
Evidence: mixed

Majority RoB: **No concerns**
Average RoB: **No concerns**
Highest RoB: **No concerns**
NMA judgment No concerns

Comparison Suphentanil:zControl
Evidence: mixed

Majority RoB: **No concerns**
Average RoB: **No concerns**
Highest RoB: **No concerns**
NMA judgment No concerns

Comparison Fentanyl:Suphentanil
Evidence: indirect

Majority RoB:	**No concerns**
Average RoB:	**No concerns**
Highest RoB:	**No concerns**
NMA judgment	No concerns

Comparison
Evidence:Mepheridine:Suphentanil
indirect

Majority RoB:	**No concerns**
Average RoB:	**No concerns**
Highest RoB:	**No concerns**
NMA judgment	No concerns

UNIVERSITÄT BERN
(https://www.ispm.unibe.ch)Institute of Social and Preventive Medicine (ISPM)

Campbell Collaboration
(https://www.campbellcollaboration.org)

Cochrane
(http://www.cochrane.org)

CINeMA is distributed, in the hope that it will be useful but without any warranty, under the AGPLv3 (LICENSE) license. By using CINeMA you accept the following DISCLAIMER (DISCLAIMER)

Confidence In Network Meta Analysis - CINeMA 1.9.1

Reporting bias

Reset Proceed

Comparison Fentanyl:Mepheridine Evidence: mixed

Reporting bias judgment

Undetected

Comparison Fentanyl:zControl Evidence: mixed

Reporting bias judgment

Undetected

Comparison Mepheridine:zControl Evidence: mixed

Reporting bias judgment

Undetected

Comparison Suphentanil:zControl Evidence: direct

Reporting bias judgment

Undetected

Comparison Fentanyl:Suphentanil Evidence: indirect

Reporting bias judgment

Undetected

Comparison Evidence:Mepheridine:Suphentanil indirect

Reporting bias judgment

Undetected

UNIVERSITÄT BERN (https://www.ispm.unibe.ch) **Institute of Social and Preventive Medicine (ISPM)**

(https://www.campbellcollaboration.org) (http://www.cochrane.org/)

CINeMA is distributed, in the hope that it will be useful but without any warranty, under the GPL v3 (LICENSE) license. By using CINeMA you accept the following **DISCLAIMER (DISCLAIMER)**

Confidence In Network Meta Analysis - CINeMA

1.9.1

Indirectness

21 total studies

13 : low **8** : moderate

Indirectness contributions

The bar chart shows the contributions of each study to the network estimate

Save Chart

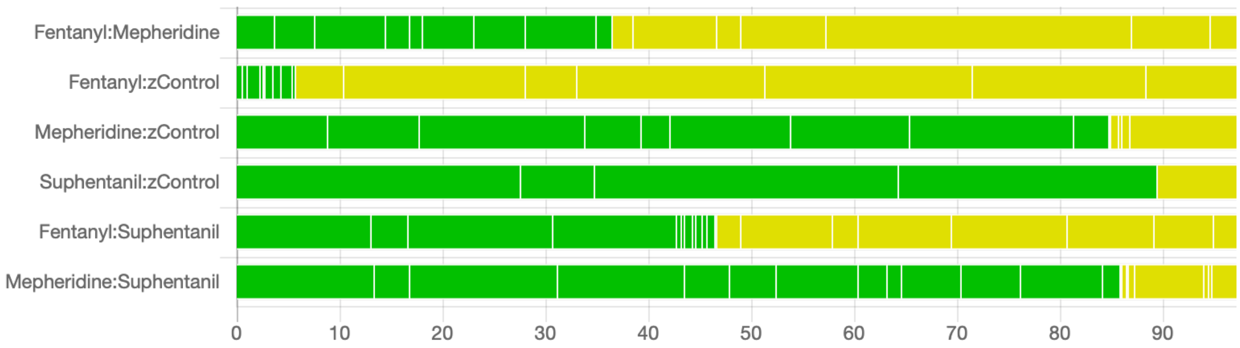

Selected rule: Average

Reset Proceed

Comparison Fentanyl:Mepheridine
Evidence: mixed

Majority:	**Some concerns**
Average:	**Some concerns**
Highest:	**Some concerns**
NMA judgment	Some concerns

Comparison Fentanyl:zControl
Evidence: mixed

Majority:	**Some concerns**
Average:	**Some concerns**
Highest:	**Some concerns**
NMA judgment	Some concerns

Comparison Mepheridine:zControl
Evidence: mixed

Majority:	**No concerns**
Average:	**No concerns**
Highest:	**Some concerns**
NMA judgment	No concerns

Comparison Suphentanil:zControl
Evidence: mixed

Majority:	**No concerns**
Average:	**No concerns**
Highest:	**Some concerns**
NMA judgment	No concerns

Comparison Fentanyl:Suphentanil Evidence: indirect

Majority:	**Some concerns**
Average:	**Some concerns**
Highest:	**Some concerns**
NMA judgment	Some concerns

Comparison Evidence:Mepheridine:Suphentanil indirect

Majority:	**No concerns**
Average:	**No concerns**
Highest:	**Some concerns**
NMA judgment	No concerns

b UNIVERSITÄT BERN
(https://www.ispm.unibe.ch)**Institute of Social and Preventive Medicine (ISPM)**

Campbell Collaboration (https://www.campbellcollaboration.org)

Cochrane (http://www.cochrane.org)

CINeMA is distributed, in the hope that it will be useful but without any warranty, under the AGPL v3 **(LICENSE)** license. By using **CINeMA** you accept the following **DISCLAIMER (DISCLAIMER)**

Confidence In Network Meta Analysis - CINeMA 1.9.1

Imprecision

Define clinically important size of effect: Odds ratio 1 Set Reset

*Relative effect estimates below **1.000** and above **1.000** are considered clinically important.*

Reset Proceed

Comparison Fentanyl:Mepheridine
Evidence: mixed

NMA estimate: **1.021**
95% Confidence interval:
Confidence interval **(0.405,2.572)** *extends into clinically important effects in **both** directions*

Imprecision judgment
Major concerns

Comparison Fentanyl:zControl
Evidence: mixed

NMA estimate: **0.173**
95% Confidence interval:
Confidence interval **(0.081,0.366)** *does not cross clinically important effect*

Imprecision judgment
No concerns

Comparison Mepheridine:zControl
Evidence: mixed

NMA estimate: **0.169**
95% Confidence interval:
Confidence interval **(0.093,0.308)** *does not cross clinically important effect*

Imprecision judgment
No concerns

Comparison Suphentanil:zControl
Evidence: mixed

NMA estimate: **0.421**
95% Confidence interval:
Confidence interval **(0.181,0.981)** *does not cross clinically important effect*

Imprecision judgment
No concerns

Comparison Fentanyl:Suphentanil
Evidence: indirect

NMA estimate: **0.410**
95% Confidence interval:
Confidence interval **(0.132,1.270)**
extends into clinically important effects in ***both*** *directions*

Imprecision judgment
Major concerns

Comparison Evidence:Mepheridine:Suphentanil indirect

NMA estimate: **0.401**
95% Confidence interval:
Confidence interval **(0.142,1.132)**
extends into clinically important effects in ***both*** *directions*

Imprecision judgment
Major concerns

b UNIVERSITÄT BERN
(https://www.ispm.unibe.ch)**Institute of Social and Preventive Medicine (ISPM)**

(https://www.campbellcollaboration.org)
(http://www.cochrane.org)

CINeMA is distributed, in the hope that it will be useful but without any warranty, under the **(LICENSE)** license. By using **CINeMA** you accept the following **DISCLAIMER (DISCLAIMER)**

Confidence In Network Meta Analysis - CINeMA

1.9.1

Heterogeneity

Define clinically important size of effect: Odds ratio 1 Set Reset

*Relative effect estimates below **1.000** and above **1.000** are considered clinically important.*
Importance of heterogeneity depends on the variability of effects in relation to a clinically important size of effect

Between-study variance estimates for each direct comparison along with reference intervals

The estimated value of between-study variance for the network meta-analysis is **0.412**

Reset Proceed

Comparison Fentanyl:Mepheridine
Evidence: mixed

NMA estimate: **1.021**

95% intervals for NMA estimate

Confidence interval: **(0.405,2.572)**

Prediction interval: **(0.191,5.443)**

*Confidence and prediction intervals **agree** in relation to clinically important effect*

Heterogeneity judgment

No concerns

Comparison Fentanyl:zControl
Evidence: mixed

NMA estimate: **0.173**

95% intervals for NMA estimate

Confidence interval: **(0.081,0.366)**

Prediction interval: **(0.036,0.830)**

*Confidence and prediction intervals **agree** in relation to clinically important effect*

Heterogeneity judgment

No concerns

Comparison Mepheridine:zControl
Evidence: mixed

NMA estimate: **0.169**

95% intervals for NMA estimate

Confidence interval: **(0.093,0.308)**

Prediction interval: **(0.038,0.753)**

*Confidence and prediction intervals **agree** in relation to clinically important effect*

Heterogeneity judgment

No concerns

Comparison Suphentanil:zControl
Evidence: mixed

NMA estimate: **0.421**

95% intervals for NMA estimate

Confidence interval: **(0.181,0.981)**

Prediction interval: **(0.083,2.139)**

*Prediction interval extends into clinically important effects in **both** directions*

Heterogeneity judgment

Major concerns

Comparison Fentanyl:Suphentanil
Evidence: indirect

NMA estimate: **0.410**

95% intervals for NMA estimate

Confidence interval: **(0.132,1.270)**

Prediction interval: **(0.067,2.514)**

*Confidence and prediction intervals **agree** in relation to clinically important effect*

Heterogeneity judgment

No concerns

Comparison
Evidence:Mepheridine:Suphentanil
indirect

NMA estimate: **0.401**

95% intervals for NMA estimate

Confidence interval: **(0.142,1.132)**

Prediction interval: **(0.070,2.305)**

*Confidence and prediction intervals **agree** in relation to clinically important effect*

Heterogeneity judgment

No concerns

UNIVERSITÄT BERN
(https://www.ispm.unibe.ch) **Institute of Social and Preventive Medicine (ISPM)**

Campbell Collaboration (https://www.campbellcollaboration.org)

Cochrane (http://www.cochrane.org)

CINeMA is distributed, in the hope that it will be useful but without any warranty, under the GPL v3 **(LICENSE)** license. By using **CINeMA** you accept the following **DISCLAIMER (DISCLAIMER)**

Confidence In Network Meta Analysis - CINeMA

1.9.1

Incoherence

Define clinically important size of effect: Odds ratio 1 Set Reset

Relative effect estimates below ***1.000*** *and above* ***1.000*** *are considered clinically important. Importance of Incoherence depends on the variability of direct and indirect effects in relation to a clinically important size of effect*

Global test based on a random-effects design-by-treatment interaction model

χ^2 statistic: 0.336 (2 degrees of freedom), P value: 0.846

Local tests: Separating indirect from direct evidence

Reset Proceed

**Comparison**	**Fentanyl:Mepheridine**
**Evidence: mixed**	
NMA odds ratio:	**1.021(0.405,2.572)**
Direct odds ratio:	**1.714(0.250,11.732)**
Indirect odds ratio:	**0.874(0.304,2.506)**
**Inconsistency measures**	
Ratio of odds ratios:	**1.962(0.219,17.591)**
P value:	**0.547**
Incoherence judgment	No concerns

**Comparison**	**Fentanyl:zControl**
**Evidence: mixed**	
NMA odds ratio:	**0.173(0.081,0.366)**
Direct odds ratio:	**0.166(0.077,0.358)**
Indirect odds ratio:	**0.410(0.010,16.172)**
**Inconsistency measures**	
Ratio of odds ratios:	**0.405(0.009,17.319)**
P value:	**0.637**
Incoherence judgment	No concerns

Comparison	Mepheridine:zControl
**Evidence: mixed**	
NMA odds ratio:	**0.169(0.093,0.308)**
Direct odds ratio:	**0.173(0.095,0.318)**
Indirect odds ratio:	**0.056(0.001,3.162)**
**Inconsistency measures**	
Ratio of odds ratios:	**3.119(0.052,185.694)**
P value:	**0.585**
Incoherence judgment	No concerns

Comparison	Suphentanil:zControl
**Evidence: direct**	
Direct odds ratio:	**0.421(0.181,0.981)**
**Inconsistency measures:** Not applicable	
Incoherence judgment	No concerns

Comparison	Fentanyl:Suphentanil
**Evidence: indirect**	
Indirect odds ratio:	**0.410(0.132,1.270)**
**Inconsistency measures:** Not applicable	
Incoherence judgment	No concerns

Comparison	Mepheridine:Suphentanil
**Evidence: indirect**	
Indirect odds ratio:	**0.401(0.142,1.132)**
**Inconsistency measures:** Not applicable	
Incoherence judgment	No concerns

UNIVERSITÄT BERN (https://www.ispm.unibe.ch) **Institute of Social and Preventive Medicine (ISPM)**

Campbell Collaboration (https://www.campbellcollaboration.org)

Cochrane (http://www.cochrane.org)

CINeMA is distributed, in the hope that it will be useful but without any warranty, under the GPL v3 **(LICENSE)** license. By using **CINeMA** you accept the following **DISCLAIMER (DISCLAIMER)**

Confidence In Network Meta Analysis - CINeMA

1.9.1

Shivering

Download Report Reset

Comparison	Number of Studies	Within-study bias	Reporting bias	Indirectness	Imprecision	Heterogeneity	Incoherence	Confidence rating
Mixed evidence								
**Fentanyl vs Mepheridine**	1	No concerns	Undetected	Some concerns	Major concerns	No concerns	No concerns	Low
**Fentanyl vs zControl**	7	No concerns	Undetected	Some concerns	No concerns	No concerns	No concerns	Low
**Mepheridine vs zControl**	10	No concerns	Undetected	No concerns	No concerns	No concerns	No concerns	Moderate
**Suphentanil vs zControl**	5	No concerns	Undetected	No concerns	No concerns	Major concerns	No concerns	Low
Indirect evidence								
**Fentanyl vs Suphentanil**	--	No concerns	Undetected	Some concerns	Major concerns	No concerns	No concerns	Low
**Mepheridine vs Suphentanil**	--	No concerns	Undetected	No concerns	Major concerns	No concerns	No concerns	Low

UNIVERSITÄT BERN
(https://www.ispm.unibe.ch)Institute of Social and Preventive Medicine (ISPM)

Campbell Collaboration (https://www.campbellcollaboration.org)

Cochrane (http://www.cochrane.org)

CINeMA is distributed, in the hope that it will be useful but without any warranty, under the AGPL (LICENSE) license. By using CINeMA you accept the following DISCLAIMER (DISCLAIMER)

Confidence In Network Meta Analysis - CINeMA 1.9.1
